# Detection of *vapN* in *Rhodococcus equi* isolates cultured from humans

**DOI:** 10.1371/journal.pone.0190829

**Published:** 2018-01-04

**Authors:** Laura K. Bryan, Ellen Ruth Alexander, Sara D. Lawhon, Noah D. Cohen

**Affiliations:** 1 Department of Veterinary Pathobiology, College of Veterinary Medicine & Biomedical Sciences, Texas A&M University, College Station, Texas, United States of America; 2 Department of Large Animal Clinical Sciences, College of Veterinary Medicine & Biomedical Sciences, Texas A&M University, College Station, Texas, United States of America; University of Parma, ITALY

## Abstract

*Rhodococcus equi* can cause severe infections in people, particularly in immunocompromised individuals. The *R*. *equi* virulence plasmids (*vap*) encoding *vapA* and *vapB* are linked to development of infections in domestic animals. Recently, a novel virulence plasmid, *vapN*, was identified in isolates cultured from cattle, but its prevalence or significance in human *R*. *equi* infections has not been extensively studied. To determine the prevalence of *vapN* in a diverse collection of human-derived isolates from different countries, 65 *R*. *equi* isolates collected by various institutions from 1984 to 2002 were screened for the presence of *vapN* and other virulence plasmids through polymerase chain reaction (PCR) using redesigned primer sets. Of the isolates that carried plasmids, 43% (16/37) were *vapN*-positive and fewer were *vapB* or *vapA*-positive (30 and 16%, respectively). This is the first report of *vapN* carriage in *R*. *equi* isolated from human infections. One isolate (H-30) carried *vapN* but did not amplify the conjugal plasmid transfer gene *traA* associated with carriage of *vap*, which could be explained by sequence variation within the *traA* gene. Another isolate (H-55) amplified *traA*, but did not amplify *vapA*, *B*, or *N* (*traA*^+^ *vapABN*^-^) with previously described primer sets or those developed for this study. The H-55 *traA* sequence had 98% identity to *traA* sequences in *vapA* plasmids, which suggests that it may carry a variant of previously characterized virulence plasmids or a novel virulence plasmid. Carriage of *vapN* in *R*. *equi* isolates derived from people is not uncommon and more research is needed to determine its significance in the epidemiology and pathogenesis of human *R*. *equi* infections.

## Introduction

*Rhodococcus equi* is a Gram-positive, facultative intracellular coccobacillus that causes infection in humans and a wide variety of animals [1]. *R*. *equi* infections are often opportunistic and commonly occur in immunocompromised individuals, with most reports in organ transplant recipients, hematopoietic cancer patients, or in people infected with human immunodeficiency virus (HIV) that progressed to acquired immunodeficiency syndrome (AIDS) [2, 3]. The first documented case of *R*. *equi* (then known as *Corynebacterium equi*) infection in a person occurred in an adult male receiving immunosuppressive therapy in 1966 [4], and the bacterium was first identified in an AIDS patient in 1983 [5]. Cavitating pulmonary abscesses, bacteremia, and lymphadenitis are the most common lesions associated with *R*. *equi* infection in people [2, 3], with extrapulmonary infections predominating in immunocompetent patients [6].

Virulence associated protein (*vap*) genes are encoded on circular and linear plasmids within large, horizontally acquired pathogenicity islands (PAI) [1]. The *vap* genes facilitate *R*. *equi* intra-macrophage survival and are associated with severe infections in animals [1, 7, 8]. To date, three *R*. *equi vap* plasmids have been identified: *vapA*, *vapB*, and *vapN* [8–11]. The *vapA* and *vapB* carrying strains are classified as virulent and intermediately virulent, respectively, based on mouse pathogenicity studies [11]. Isolates that carry large, circular 80–90 kb plasmids encoding *vapA* are associated with severe pulmonary and systemic infections in foals [10]. Intermediately virulent strains carrying 20 kb plasmids encoding *vapB* are commonly isolated from the lymph nodes of swine [1, 12] and are not as pathogenic in horses when compared to *vapA*-positive strains [13]. The novel linear virulence plasmid encoding *vapN* was discovered in 2015 and is primarily associated with isolates from the lymph nodes of cattle and other ruminants [1, 8, 14]. Carriage of specific *vap* plasmids is highly associated with animal host species, which suggests that *vap* plasmids facilitate host-adaptation [1], but equine *R*. *equi* isolates carrying *vapA* and porcine *vapB* isolates can survive in the macrophages of different animal species [15]. The *Rhodococcus* spp. *traA* gene encodes a conserved relaxase that initiates transfer of virulence plasmids during conjugation, and has been identified in *R*. *equi* isolates that carry *vap* plasmids [12, 16]. Prior to the development of a typing strategy incorporating the presence of the *traA* gene, strains that did not carry *vapA* or *vapB* were classified as avirulent [11, 12]. Subsequent analysis of previously typed avirulent strains revealed that many did carry the *traA* gene but not the *vapA* or *vapB* plasmids (*traA*+ *vapAB*- genotype) [12].

Human *R*. *equi* infections are thought to be opportunistic and zoonotic transmission from contact with animals or contaminated meat or other animal byproducts has been proposed [1, 8, 14]. Most human isolates either carry *vapB* or are *traA*+ *vapAB*-, while *vapA* carriage is less frequently observed [11, 12, 14]. In a report of 74 clinical isolates from patients in Brazil, 2 isolates carrying *vapN* were recovered from the lymph nodes of AIDS patients [14]. It is unknown if the *traA*^+^ *vapAB*^-^ genotype mentioned in early reports can be attributed solely to *vapN* carriage, and the only report examining human isolates did not screen for the presence of *traA* in the avirulent samples [14]. The *vap* plasmids share a high degree of sequence similarity in the housekeeping backbone region of the plasmid, but the regions encoding the *vap* genes are distinct [1]. However, there is sequence variation within PAI in inter-genic regions, most likely due to single nucleotide polymorphisms (SNP) or nucleotide insertion/deletion (INDEL) events [1], which can contribute to non-specific amplification of polymerase chain reaction (PCR) products designed to flank *vap* coding regions. Purification and typing of *vap* plasmids using traditional methods, such as restriction enzyme digestion, are time consuming and labor intensive when compared to PCR methods that utilize genomic DNA (gDNA) [12]. The goals of this study were to develop specific PCR primers for detection of *vap* plasmids that could be used with gDNA and to screen a historic database of human-derived *R*. *equi* isolates for *vap* plasmids to determine prevalence of *vapN* carriage.

## Materials and methods

### Collection demographics

A collection of 65 *R*. *equi* clinical isolates cultured from 62 people from 1984–2002 was used in the PCR analyses (S1 Table). One third of the isolates were originally submitted to the United States Centers for Disease Control (CDC; Atlanta, Georgia, USA) and were collected from patients in Arkansas (n = 1), Brazil (n = 2), Canada (n = 1), Connecticut (n = 2), Delaware (n = 1), Florida (n = 1), France (n = 1), India (n = 1), Italy (n = 3), North Carolina (n = 1), Ohio (n = 2), Oklahoma (n = 1), Pennsylvania (n = 1), Puerto Rico (n = 1), Spain (n = 1), Tennessee (n = 1), and Virginia (n = 1). The remaining isolates came from the California Department of Health Services (CDHS; n = 10), Mayo Clinic (Rochester, Minnesota; n = 10), Texas Department of State Health Services (n = 17), and the University of Texas Health Science Center at Tyler (n = 6). The *R*. *equi* isolates were shared with Texas A&M University by the respective receiving institutions beginning in 2001; all isolates were residual diagnostic specimens and no identifying information of the patients (including sex or age) was recorded at time of transfer to Texas A&M.

Patient 1 from Fort Worth and patient 12 from San Antonio, Texas contributed multiple *R*. *equi* isolates (3 and 2 isolates, respectively) collected at different time points. Of the 27 specimens with culture-site information, 13 were from blood, 8 from lavage or biopsy of respiratory tissues, 4 from sputum, 1 from peritoneal fluid, and 1 from a neck abscess. One of the blood isolates came from a person with an infected tooth extraction site and another came from a patient with end-stage renal disease. Sixteen of the isolates were recorded as coming from HIV/AIDS patients, 1 came from a cancer patient, and 2 were from heart or coronary artery transplant patients.

### Bacterial culture and molecular techniques

#### Culture and DNA extraction

Isolates were preserved at -80°C on beads in CryoCare^™^ Bacteria Preservers tubes (Key Scientific Products, Stamford, TX, USA) after initial culture and transfer of the collection. Isolates were grown for 24 hours in modified NANAT selective medium [17] and then struck onto modified NANAT agar for incubation at 37°C for 48 hours. For DNA extraction, a colony was inoculated into 50 μl of *Mycobacterium* spp. DNA extraction medium [18], and the solution was boiled for 10 minutes in a PCR thermal cycler. The samples were briefly centrifuged to pellet sediment, and 2 μL of the supernatant was used as template for the PCR analyses. Genomic DNA was extracted from isolates H-30 and H-55 using a *DNeasy*® Blood & Tissue Kit (Qiagen, Germantown, MD, USA) per the manufacturer’s recommendations for Gram-positive bacteria.

#### PCR assay and validation

New PCR primer sets (Table 1) for individual detection of *vapA*, *vapB*, and *vapN* were designed with Primer-BLAST [19] and cross-checked with the Nucleotide Basic Local Alignment Search Tool (BLASTN) for sequence similarity with *Rhodococcus* spp. chromosomal DNA and between all of the *vap* plasmid sequences deposited in GenBank as of August 15, 2017. Primers amplifying internal regions of the *vap* genes or PAI-type conserved flanking regions were selected to minimize non-specific products. The PCR results for the new primer set were compared with the results from previously published primer sets for detection of *vap* genes [12, 20]. A PCR using the *R*. *equi*-specific cholesterol oxidase gene *choE* was used to ensure correct speciation and adequate DNA extraction for each isolate [21]. The following control strains were used for each PCR assay: ATCC^®^ 33701^™^ (*vapA*-positive), clinical porcine isolate EIDL 99–213 (*vapB*-positive), clinical canine isolate TAMU 49–33 (*vapN*-positive) [20], and ATCC^®^ 6939^™^ (avirulent genotype). PCR reagent concentrations were calculated according to the manufacturer’s recommendations (AmpliTaq^®^ DNA polymerase; ThermoFisher Scientific, USA). PCR conditions consisted of an initial denaturing step at 95°C for 5 minutes, 30 cycles of 95°C for 30 seconds, annealing at 56°C for 30 seconds, and extension at 72°C for 2 minutes, followed by a final extension step of 72°C for 10 minutes. Amplicons were visualized on a 1% agarose gel stained with GelRed (Biotium, USA) and electrophoresed at 115V for 30 minutes.

**Table 1:** Oligonucleotide primer sequences.

Gene	Primer sequence (5’ → 3’)	Amplicon size (bp)	Reference
*choE*	F- GTCAACAACATCGACCAGGCG	959	[21]
	R- CGAGCCGTCCACGACGTACAG		
*traA*	F- AGAGTTCATGCGTGACAACG	959	[12]
	R- GTCCACAGGTCACCGTTCTT		
*vapA*	F- AGACTCTTCACAAGACGGTTTCT	334	This study
	R- TCGCCATCGAAGACCTTTCCTT		
*vapB*	F- CTTCTTAAGGATGGGGCAGG	485	This study
	R-GGCTACCTTCAGCCTGCTAT		
*vapN*	F-GGTACTGCAGGCAACTGCTA	425	This study
	R-GAGCTGCTACTACCGTGGTC		

New primer sets were designed for the *vap* genes and used in conjunction with previous typing schemes. Sequences are given in the 5’ to 3’ direction.

For validation of the PCR primer sets, an amplicon from a randomly selected isolate positive for *vapA* (H-56), *vapB* (H-32), or *vapN* (H-5) was purified using a NucleoSpin Gel and PCR Clean-up kit (Macherey-Nagel, Düren, Germany) and submitted for Sanger sequencing (Eton Biosciences, San Diego, CA, USA). The *traA* and *vapN* amplicons from H-55 and H-30, respectively, and the aberrant 99–213 *vapN* band were also sequenced. The forward and reverse sequences were trimmed, aligned, and consensus sequence generated using Geneious R10 software (Biomatters Limited, Auckland, New Zealand); the sequences were queried against GenBank accessions using BLASTN. The partial *traA* sequence from H-55 was deposited in GenBank under accession number MF680842 (https://www.ncbi.nlm.nih.gov/nuccore/MF680842).

### Statistical analyses

Statistical analyses were performed with JMP Pro 12 (SAS Institute Inc., Cary, North Carolina, USA). Contingency tables were used for categorical variables, and a chi-squared test (CST) or Fisher’s exact test (FET) was used when expected values for a cell were less than five. A significance level of p < 0.05 was used for all analyses.

## Results

**Fig 1:**
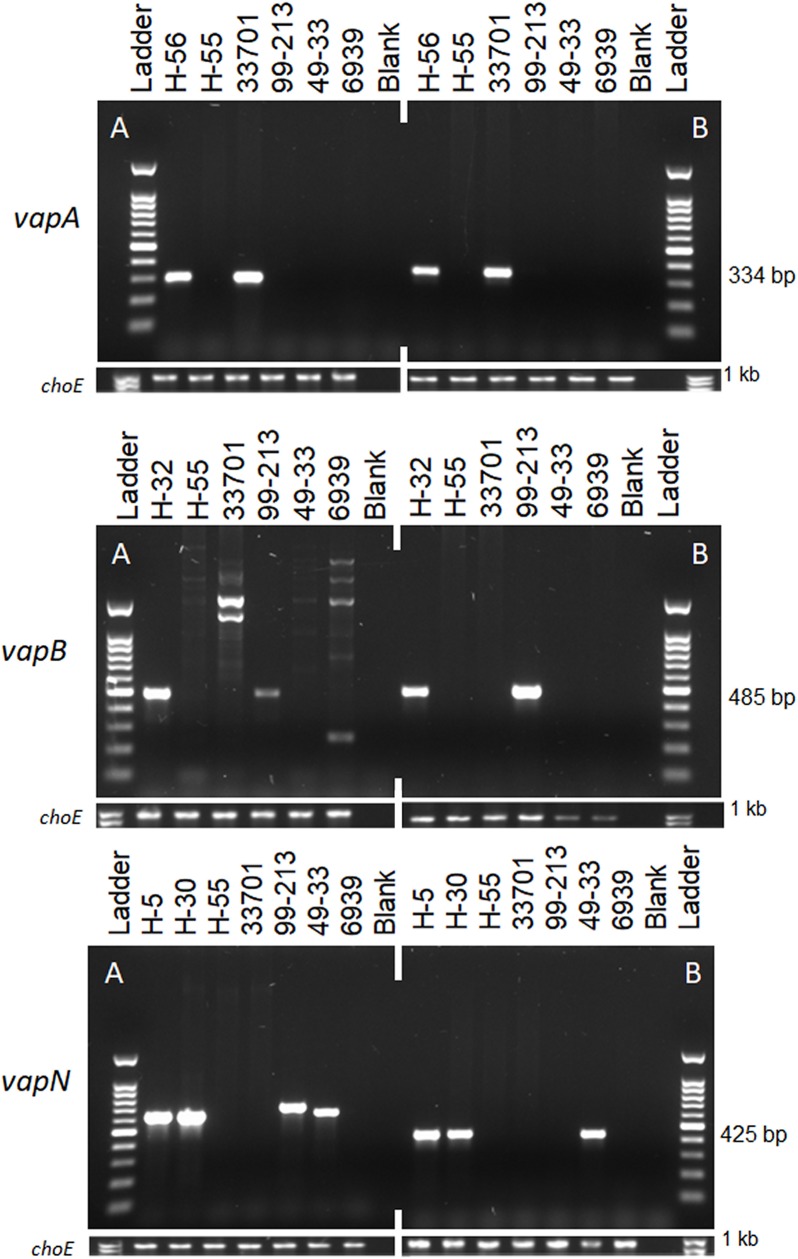
**Agarose gel electrophoresis comparison of previously published primer sets (A) and those designed for this study (B) for the *vapA*, *vapB*, *and vapN* genes.** The expected amplicon sizes for the A sets are *vapA* (286 bp), *vapB* (477 bp), and *vapN* (625 bp). Reactions for *vapA* and *vapB* were performed individually and not in multiplex as originally described [12]. The human isolates depicted are H-5 (*traA+ vapN+ vapAB-*), H-30 (*traA- vapN+ vapAB-*), H-32 (*traA+ vapB+ vapAN-*), H-55 (*traA+ vapABN-*), and H-56 (*traA+ vapA+ vapBN-*). The control strains used were ATCC^®^ 33701^™^ (*vapA*-positive), clinical porcine isolate EIDL 99–213 (*vapB*-positive), clinical canine isolate TAMU 49–33 (*vapN*-positive), and ATCC^®^ 6939^™^ (avirulent genotype). The corresponding *choE* DNA amplification control band for each reaction is below the panels. The DNA ladder used was Ready-to-use 100 bp ladder (Biotium; Fremont, CA) with fragments from 100 to 1500 bp. Both *vapA* primer sets were specific for *vapA*. There is non-specific amplification in the non-*vapB-*positive and non-*vapN*-positive isolates with the previous primer sets. The primer sets developed for this study (B) amplified a single product.

The new primer sets were specific and amplified single products for each gene investigated, and results were similar whether using gDNA extracted through the boil-plate method or commercial kit. A comparison of the PCR results (Fig 1) for select isolates using the previously published multiplex *vapAB* and *vapN* primer sets (A) and those developed for this study (B) revealed non-specific amplification of products in the previously described primer sets. Multiple bands were observed for the previously published *vapB* primers [12] in isolates that did not carry the *vapB* plasmid. The *vapB* control strain 99–213 and the majority of the *vapB*-positive human isolates amplified a non-specific band of approximately 700 bp with the previously published *vapN* primer set [20]. Sequencing of the aberrant band in 99–213 revealed that it was a region of the *vapB* gene (99% identity to KX443407.1) and was not similar to the intended *vapN* gene. The aberrant *vapN* band observed in 99–213 did not occur in the *vapN* primer set designed for this study.

**Fig 2:**
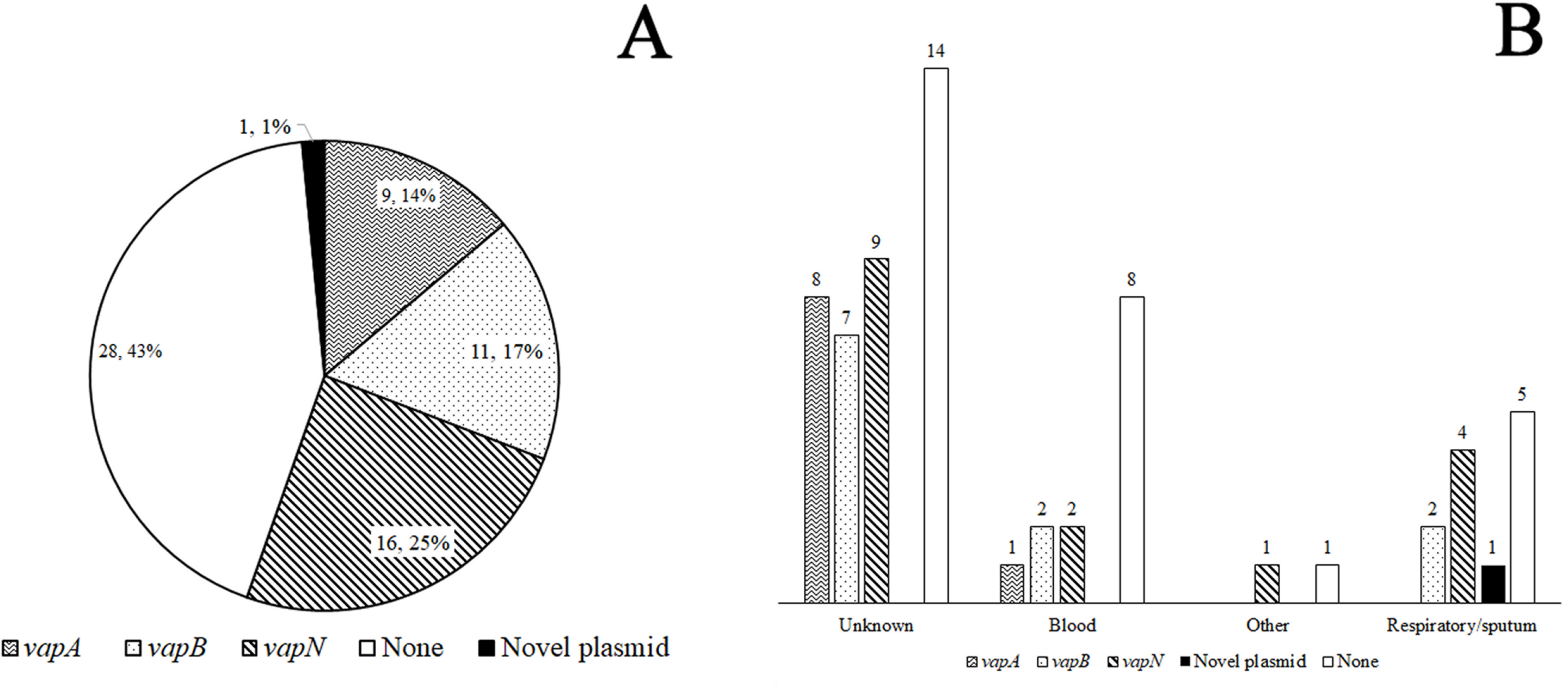
**Carriage prevalence of *vapA*, *vapB* and *vapN* in 65 human *R*. *equi* isolates (A) and *vap* plasmid prevalence based on culture site (B).** The Other category includes peritoneal fluid and neck abscess specimens.

All of the isolates were *choE*-positive, indicating that they were correctly speciated as *R*. *equi*. Queries using BLASTN for each of the selected amplicons for primer validation revealed 100, 99, and 100% identity with respective *vapA*, *vapB*, and *vapN* sequences deposited in GenBank (KX443405.1, KX443407.1, and KX443401.1). The majority (57%; 37/65) of the isolates carried virulence plasmids (Fig 2A), with 25% (16/65) positive for *vapN*, 17% (11/65) for *vapB*, and 14% (9/65) for *vapA* across all culture sites (Fig 2B). Of the 16 isolates recorded as coming from HIV-positive or AIDS patients, 8 were *traA*-negative (avirulent), 5 carried *vapN*, 2 *vapB*, and 1 *vapA*. One isolate (H-55) cultured in 2000 from a respiratory infection in patient 52 carried *traA* but did not amplify *vapA*, *vapB*, or *vapN* (*traA*^*+*^ *vapABN*^-^) with previously described primer sets or those developed for this study. The H-55 *traA* sequence had 98% sequence identity with a segment of *traA* in multiple *vapA* plasmid accessions in GenBank (*i*.*e*., KX443388.1, HM114217.1, etc.). Another isolate (H-30) cultured in 1988 from an AIDS patient in California (patient 27), carried *vapN* with 99% sequence identity to *vapN* (KX443401.1) in GenBank but did not amplify *traA* (*traA- vapN+ vapAB-*). All three samples from patient 1 carried *vapB*, and both of the isolates collected from patient 12 carried *vapN*.

The earliest instance of *vapN* carriage in the collection was an isolate cultured from an AIDS patient in 1985 (patient 30) by the CDHS and the last instance was in a 2002 culture from the Mayo Clinic (patient 59). *VapN*-positive isolates were also collected from 5 other patients in California, 6 patients in Texas, 1 in Minnesota, an HIV-positive patient in Brazil with a neck abscess, and from a coronary artery transplant patient in Puerto Rico that was reported to be HIV-negative. Of the 9 *vapA*-positive isolates, 6 came from patients in Texas, 2 in Minnesota, and 1 in Delaware; the only instance of *vapA* among the isolates with culture site information was in an HIV-positive bacteremia patient. Two bacteremia patients were *vapB*-positive, 1 in Canada and the other in California secondary to tooth root extraction complications. Additional *vapB*-positive isolates came from 6 patients in Texas and single patients in Virginia, Spain, and Italy. Carriage of *vap* plasmids did not differ significantly between the isolates with known culture site information (p = 0.50; FET) or for the subset of patients reported as having HIV/AIDS (p = 0.28; CST). The carriage of *vapA*, *vapB*, or *vapN* did not differ by isolate collection year (p = 0.77, 0.50, and 0.08, respectively; FET).

## Discussion

The overall prevalence of *vapN* carriage within the collection was 25% (16/65), and *vapN* was encountered in 43% (16/37) of all isolates that had plasmids. This is higher than the 2.7% (2/74) *vapN* prevalence rate reported in patients from Brazil [14]. Isolates carrying *vapN* were found in the United States, Brazil, and Puerto Rico. Similar to the previous report [14], the 1992 Brazilian *vapN* isolate in our collection was from a HIV-positive patient with a neck abscess, which may have originated in a cervical lymph node. The *vapN* plasmid has been detected in *R*. *equi* isolates from cattle, sheep, goats, a dog, and human patients with AIDS [1, 8, 14, 20]. To the authors’ knowledge, this is the first confirmed report of *vapN* carriage in *R*. *equi* cultured from patients outside of Brazil and from a HIV-negative, transplant patient. The majority (73%; 27/37) of the plasmid-positive isolates carried either *vapN* or *vapB* with fewer *vapA*-positive strains, which is similar to the *vapA* and *vapB* plasmid prevalence distributions reported in previous studies with human isolates [7, 12, 14]. Since no demographic information was available for the collection, it is unknown if patients infected with *vapN* -positive *R*. *equi* strains were directly exposed to livestock, contaminated animal products or lived in rural environments where the bacterium might be found in soil or on fomites in contact with animals.

In a previous report that typed isolates based on *traA* carriage but was published before the discovery of *vapN*, most *R*. *equi* isolates cultured from people either carried *vapB* or had the *traA+ vapAB-* genotype [12]. Based on our findings, it is likely that many strains categorized as *traA+ vapAB-* actually carry *vapN*. However, the single *traA+ vapABN-* isolate (H-55) discovered in our collection suggests that there may be sequence variation amongst the described *vap* plasmids or perhaps an entirely new virulence plasmid within the population, as the relatively conserved *traA* sequence was similar but not identical to other described *vap* plasmids. While carriage of *traA* in isolates with *vap* plasmids is strongly associated [12], 1 isolate (H-30) in this study was *vapN*-positive but did not amplify *traA* (*traA- vapN+ vapAB-*). The most likely explanation for this observation is that SNP or INDEL in H-30 at the *traA* primer annealing sites prevented amplification of *traA*. In light of this finding, PCR for specific *vap* genes should be done on both *traA*-positive and -negative isolates to confirm genotype.

## Conclusions

While not a substitute for full characterization of *vap* plasmids, the PCR primers developed for this study amplify the respective *vap* genes specifically and can be used with different methods of gDNA extraction for efficient detection of *vap* plasmids. Based on the findings, current *R*. *equi* clinical isolates and previously typed avirulent (or *traA-*negative) strains in historic clinical collections should be checked for *vapN* to ensure that they were not misidentified. Further study is warranted to gauge the significance of *vapN* in the pathogenesis of human *R*. *equi* infections.

## Supporting information

S1 Table Isolate information and virulence plasmid genotype for 65 human-derived *R*. *equi* samples. A 1 denotes amplification of the specified gene and a 0 signifies no amplification. CDC = Centers for Disease Control; CDHS = California Department of Health Services; ID = identification; MC = Mayo Clinic; TDHS = Texas Department of Health Services; UTHSC = University of Texas Health Science Center.
(DOCX)
