# Correction: Detection of *vapN* in *Rhodococcus equi* isolates cultured from humans

**DOI:** 10.1371/journal.pone.0235719

**Published:** 2020-06-30

**Authors:** Laura K. Bryan, Ellen Ruth Alexander, Sara D. Lawhon, Noah D. Cohen

Following the publication of this article [1], a reader noticed the sixth sentence of the Abstract and the fifth sentence of the first paragraph of the Discussion are incorrect. The *vapN* carriage of *R*. *equi* isolated from human infections from Brazil was reported during the revision process of this article [2] and an isolate from Japan has been previously identified [3]. The two sentences in question were not corrected between revisions. Please see the correct sentences here:

There is an error in the sixth sentence of the Abstract. The correct sentence is: This is the first report of *vapN* carriage in *R*. *equi* isolated from human infections in the United States.

There is an error in the fifth sentence of the first paragraph of the Discussion. The correct sentence is: To the authors' knowledge, this is the first confirmed report of *vapN* carriage in *R*. *equi* cultured from patients in the United States and from a HIV-negative, transplant patient.
