# Supplementary material for: Detection of vapN in Rhodococcus equi isolates cultured from humans
Source: PLoS One. 2018 Jan 4;13(1):e0190829. doi: 10.1371/journal.pone.0190829 (PMC5754133; doi:10.1371/journal.pone.0190829)
Supplement: S1 Table — A 1 denotes amplification of the specified gene and a 0 signifies no amplification. CDC = Centers for Disease Control; CDHS = California Department of Health Services; ID = identification; MC = Mayo Clinic; TDHS = Texas Department of Health Services; UTHSC = University of Texas Health Science Center. (DOCX) [file pone.0190829.s001.docx]

**S1 Table:** **Isolate information and virulence plasmid genotype for 65 human-derived *R. equi* samples.** A 1 denotes amplification of the specified gene and a 0 signifies no amplification. CDC = Centers for Disease Control; CDHS = California Department of Health Services; ID = identification; MC = Mayo Clinic; TDHS = Texas Department of Health Services; UTHSC = University of Texas Health Science Center

| Isolate ID | Patient ID | Year collected | Culture site | Receiving institution | Location | Notes | *traA* | *vapA* | *vapB* | *vapN* | *choE* | Genotype |
| --- | --- | --- | --- | --- | --- | --- | --- | --- | --- | --- | --- | --- |
| H-1 | 1 | January, 1999 | unknown | TDHS | Fort Worth, Texas, USA |  | 1 | 0 | 1 | 0 | 1 | *traA+ vapB+ vapAN-* |
| H-2 | 2 | 1999 | unknown | TDHS | Austin, Texas, USA |  | 0 | 0 | 0 | 0 | 1 | *traA- vapABN-* |
| H-3 | 3 | 1998 | unknown | TDHS | Laredo, Texas, USA |  | 1 | 0 | 0 | 1 | 1 | *traA+ vapN+ vapAB-* |
| H-4 | 4 | 1999 | unknown | TDHS | San Antonio, Texas, USA |  | 1 | 0 | 0 | 1 | 1 | *traA+ vapN+ vapAB-* |
| H-5 | 5 | 1999 | unknown | TDHS | San Antonio, Texas, USA |  | 1 | 0 | 0 | 1 | 1 | *traA+ vapN+ vapAB-* |
| H-6 | 6 | 1999 | unknown | TDHS | Dallas, Texas, USA |  | 0 | 0 | 0 | 0 | 1 | *traA- vapABN-* |
| H-7 | 1 | April, 1999 | unknown | TDHS | Fort Worth, Texas, USA |  | 1 | 0 | 1 | 0 | 1 | *traA+ vapB+ vapAN-* |
| H-8 | 7 | 1999 | unknown | TDHS | Lubbock, Texas, USA |  | 1 | 1 | 0 | 0 | 1 | *traA+ vapA+ vapBN-* |
| H-9 | 8 | 1999 | unknown | TDHS | Dallas, Texas, USA |  | 1 | 1 | 0 | 0 | 1 | *traA+ vapA+ vapBN-* |
| H-10 | 1 | April, 1999 | unknown | TDHS | Fort Worth, Texas, USA |  | 1 | 0 | 1 | 0 | 1 | *traA+ vapB+ vapAN-* |
| H-11 | 9 | 1999 | unknown | TDHS | Fort Worth, Texas, USA |  | 1 | 0 | 1 | 0 | 1 | *traA+ vapB+ vapAN-* |
| H-12 | 10 | 2001 | unknown | TDHS | Corpus Christi, Texas, USA |  | 1 | 0 | 0 | 1 | 1 | *traA+ vapN+ vapAB-* |
| H-13 | 11 | 2001 | unknown | TDHS | Denton, Texas, USA |  | 1 | 1 | 0 | 0 | 1 | *traA+ vapA+ vapBN-* |
| H-14 | 12 | 2001 | unknown | TDHS | San Antonio, Texas, USA |  | 1 | 0 | 0 | 1 | 1 | *traA+ vapN+ vapAB-* |
| H-15 | 12 | 2001 | unknown | TDHS | San Antonio, Texas, USA |  | 1 | 0 | 0 | 1 | 1 | *traA+ vapN+ vapAB-* |
| H-16 | 13 | 2001 | unknown | TDHS | Galveston, Texas, USA |  | 0 | 0 | 0 | 0 | 1 | *traA- vapABN-* |
| H-17 | 14 | 2001 | unknown | TDHS | Galveston, Texas, USA |  | 0 | 0 | 0 | 0 | 1 | *traA- vapABN-* |
| H-18 | 15 | 2001 | unknown | UTHSC | Tyler, Texas, USA |  | 1 | 0 | 1 | 0 | 1 | *traA+ vapB+ vapAN-* |
| H-19 | 16 | 2001 | unknown | UTHSC | Tyler, Texas, USA |  | 1 | 1 | 0 | 0 | 1 | *traA+ vapA+ vapBN-* |
| H-20 | 17 | 2001 | unknown | UTHSC | Tyler, Texas, USA |  | 1 | 1 | 0 | 0 | 1 | *traA+ vapA+ vapBN-* |
| H-21 | 18 | 2001 | unknown | UTHSC | Tyler, Texas, USA |  | 1 | 0 | 0 | 1 | 1 | *traA+ vapN+ vapAB-* |
| Isolate ID | Patient ID | Year collected | Culture site | Receiving institution | Location | Notes | *traA* | *vapA* | *vapB* | *vapN* | *choE* | Genotype |
| H-22 | 19 | 2001 | unknown | UTHSC | Tyler, Texas, USA |  | 1 | 0 | 1 | 0 | 1 | *traA+ vapB+ vapAN-* |
| H-23 | 20 | 2001 | unknown | UTHSC | Tyler, Texas, USA |  | 1 | 1 | 0 | 0 | 1 | *traA+ vapA+ vapBN-* |
| H-24 | 21 | 1998 | respiratory | CDHS | California, USA | AIDS, *Pneumocystis* pneumonia | 1 | 0 | 0 | 1 | 1 | *traA+ vapN+ vapAB-* |
| H-25 | 22 | 1997 | blood | CDHS | California, USA | End stage renal disease | 0 | 0 | 0 | 0 | 1 | *traA- vapABN-* |
| H-26 | 23 | 1997 | unknown | CDHS | California, USA |  | 0 | 0 | 0 | 0 | 1 | *traA- vapABN-* |
| H-27 | 24 | 1996 | sputum | CDHS | California, USA |  | 1 | 0 | 0 | 1 | 1 | *traA+ vapN+ vapAB-* |
| H-28 | 25 | 1992 | blood | CDHS | California, USA |  | 0 | 0 | 0 | 0 | 1 | *traA- vapABN-* |
| H-29 | 26 | 1991 | respiratory | CDHS | California, USA | Lung abscesses | 1 | 0 | 0 | 1 | 1 | *traA+ vapN+ vapAB-* |
| H-30 | 27 | 1988 | sputum | CDHS | California, USA | AIDS | 0 | 0 | 0 | 1 | 1 | *traA- vapN+ vapAB-* |
| H-31 | 28 | 1988 | blood | CDHS | California, USA | AIDS | 1 | 0 | 0 | 1 | 1 | *traA+ vapN+ vapAB-* |
| H-32 | 29 | 1988 | blood | CDHS | California, USA | Bacteremia secondary to tooth extraction | 1 | 0 | 1 | 0 | 1 | *traA+ vapB+ vapAN-* |
| H-33 | 30 | 1985 | blood | CDHS | California, USA | AIDS | 1 | 0 | 0 | 1 | 1 | *traA+ vapN+ vapAB-* |
| H-34 | 31 | 1989 | blood | CDC | Tennessee, USA | HIV-positive | 0 | 0 | 0 | 0 | 1 | *traA- vapABN-* |
| H-35 | 32 | 1984 | sputum | CDC | Connecticut, USA |  | 0 | 0 | 0 | 0 | 1 | *traA- vapABN-* |
| H-36 | 33 | 1989 | respiratory | CDC | Pennsylvania, USA | HIV-positive | 0 | 0 | 0 | 0 | 1 | *traA- vapABN-* |
| H-37 | 34 | 1990 | blood | CDC | Florida, USA | HIV-positive | 0 | 0 | 0 | 0 | 1 | *traA- vapABN-* |
| H-38 | 35 | 1990 | respiratory | CDC | Brazil | HIV-positive | 0 | 0 | 0 | 0 | 1 | *traA- vapABN-* |
| H-39 | 36 | 1990 | respiratory | CDC | Spain | HIV-positive | 1 | 0 | 1 | 0 | 1 | *traA+ vapB+ vapAN-* |
| H-40 | 37 | 1987 | blood | CDC | Delaware, USA | HIV-positive | 1 | 1 | 0 | 0 | 1 | *traA+ vapA+ vapBN-* |
| H-41 | 38 | 1987 | respiratory | CDC | France | HIV-positive | 0 | 0 | 0 | 0 | 1 | *traA- vapABN-* |
| H-42 | 39 | 1992 | neck abscess | CDC | Brazil | HIV-positive | 1 | 0 | 0 | 1 | 1 | *traA+ vapN+ vapAB-* |
| H-43 | 40 | 1992 | unknown | CDC | Italy | HIV-positive | 0 | 0 | 0 | 0 | 1 | *traA- vapABN-* |
| H-44 | 41 | 1992 | unknown | CDC | Italy | HIV-positive | 0 | 0 | 0 | 0 | 1 | *traA- vapABN-* |
| H-45 | 42 | 1992 | unknown | CDC | Italy | HIV-positive | 1 | 0 | 1 | 0 | 1 | *traA+ vapB+ vapAN-* |
| H-46 | 43 | 1996 | respiratory | CDC | Virginia, USA |  | 1 | 0 | 1 | 0 | 1 | *traA+ vapB+ vapAN-* |
| H-47 | 44 | 1997 | unknown | CDC | North Carolina, USA | HIV-positive | 0 | 0 | 0 | 0 | 1 | *traA- vapABN-* |
| H-48 | 45 | 1997 | blood | CDC | Arkansas, USA | Cancer | 0 | 0 | 0 | 0 | 1 | *traA- vapABN-* |
| H-49 | 46 | 1997 | sputum | CDC | Ohio, USA |  | 0 | 0 | 0 | 0 | 1 | *traA- vapABN-* |
| Isolate ID | Patient ID | Year collected | Culture site | Receiving institution | Location | Notes | *traA* | *vapA* | *vapB* | *vapN* | *choE* | Genotype |
| H-50 | 47 | 1997 | peritoneal fluid | CDC | Connecticut, USA | Heart transplant | 0 | 0 | 0 | 0 | 1 | *traA- vapABN-* |
| H-51 | 48 | 2000 | blood | CDC | Canada |  | 1 | 0 | 1 | 0 | 1 | *traA+ vapB+ vapAN-* |
| H-52 | 49 | 2000 | blood | CDC | Ohio, USA |  | 0 | 0 | 0 | 0 | 1 | *traA- vapABN-* |
| H-53 | 50 | 2000 | blood | CDC | India |  | 0 | 0 | 0 | 0 | 1 | *traA- vapABN-* |
| H-54 | 51 | 2000 | unknown | CDC | Puerto Rico | Coronary artery transplant | 1 | 0 | 0 | 1 | 1 | *traA+ vapN+ vapAB-* |
| H-55 | 52 | 2000 | respiratory | CDC | Oklahoma, USA |  | 1 | 0 | 0 | 0 | 1 | *traA+ vapABN-* |
| H-56 | 53 | 2002 | unknown | MC | Rochester, Minnesota, USA |  | 1 | 1 | 0 | 0 | 1 | *traA+ vapA+ vapBN-* |
| H-57 | 54 | 2002 | unknown | MC | Rochester, Minnesota, USA |  | 0 | 0 | 0 | 0 | 1 | *traA- vapABN-* |
| H-58 | 55 | 2002 | unknown | MC | Rochester, Minnesota, USA |  | 0 | 0 | 0 | 0 | 1 | *traA- vapABN-* |
| H-59 | 56 | 2002 | unknown | MC | Rochester, Minnesota, USA |  | 0 | 0 | 0 | 0 | 1 | *traA- vapABN-* |
| H-60 | 57 | 2002 | unknown | MC | Rochester, Minnesota, USA |  | 0 | 0 | 0 | 0 | 1 | *traA- vapABN-* |
| H-61 | 58 | 2002 | unknown | MC | Rochester, Minnesota, USA |  | 0 | 0 | 0 | 0 | 1 | *traA- vapABN-* |
| H-62 | 59 | 2002 | unknown | MC | Rochester, Minnesota, USA |  | 1 | 0 | 0 | 1 | 1 | *traA+ vapN+ vapAB-* |
| H-63 | 60 | 2002 | unknown | MC | Rochester, Minnesota, USA |  | 1 | 1 | 0 | 0 | 1 | *traA+ vapA+ vapBN-* |
| H-64 | 61 | 2002 | blood | MC | Rochester, Minnesota, USA |  | 0 | 0 | 0 | 0 | 1 | *traA- vapABN-* |
| H-65 | 62 | 2002 | unknown | MC | Rochester, Minnesota, USA |  | 0 | 0 | 0 | 0 | 1 | *traA- vapABN-* |
